# PPARα and PPARγ activation attenuates total free fatty acid and triglyceride accumulation in macrophages via the inhibition of *Fatp1* expression

**DOI:** 10.1038/s41419-018-1135-3

**Published:** 2019-01-15

**Authors:** Guozhu Ye, Han Gao, Zhichao Wang, Yi Lin, Xu Liao, Han Zhang, Yulang Chi, Huimin Zhu, Sijun Dong

**Affiliations:** 1 Center for Excellence in Regional Atmospheric Environment, Institute of Urban Environment, Chinese Academy of Sciences, 1799 Jimei Road, Xiamen, 361021 China; 2 Key Laboratory of Urban Environment and Health, Institute of Urban Environment, Chinese Academy of Sciences, 1799 Jimei Road, Xiamen, 361021 China; 3 University of Chinese Academy of Sciences, 19 Yuquan Road, Beijing, 100049 China; 4 CAS Key Laboratory of Separation Science for Analytical Chemistry, Dalian Institute of Chemical Physics, Chinese Academy of Sciences, 457 Zhongshan Road, Dalian, 116023 China

## Abstract

Lipid accumulation in macrophages interacts with microenvironment signals and accelerates diabetic atherosclerosis. However, the molecular mechanisms by which macrophage metabolism interacts with microenvironment signals during lipid accumulation are not clearly understood. Accordingly, an untargeted metabolomics approach was employed to characterize the metabolic reprogramming, and to identify potential regulatory targets related to lipid accumulation in macrophages treated with oleate, an important nutrient. The metabolomics approach revealed that multiple metabolic pathways were significantly disturbed in oleate-treated macrophages. We discovered that amino acids, nucleosides, lactate, monoacylglycerols, total free fatty acids (FFAs), and triglycerides (TGs) accumulated in oleate-treated macrophages, but these effects were effectively attenuated or even abolished by resveratrol. Notably, 1-monooleoylglycerol and 2-monooleoylglycerol showed the largest fold changes in the levels among the differential metabolites. Subsequently, we found that oleate triggered total FFA and TG accumulation in macrophages by accelerating FFA influx through the activation of *Fatp1* expression, but this effect was attenuated by resveratrol via the activation of PPARα and PPARγ signaling. We verified that the activation of PPARα and PPARγ by WY14643 and pioglitazone, respectively, attenuated oleate triggered total FFA and TG accumulation in macrophages by repressing FFA import via the suppression of *Fatp1* expression. Furthermore, the inhibition of *Fatp1* by tumor necrosis factor α alleviated oleate-induced total FFA and TG accumulation in macrophages. This study provided the first demonstration that accumulation of amino acids, nucleosides, lactate, monoacylglycerols, total FFAs, and TGs in oleate-treated macrophages is effectively attenuated or even abolished by resveratrol, and that the activation of PPARα and PPARγ attenuates oleate-induced total FFA and TG accumulation via suppression of *Fatp1* expression in macrophages. Therapeutic strategies aim to activate PPAR signaling, and to repress FFA import and triglyceride synthesis are promising approaches to reduce the risk of obesity, diabetes and atherosclerosis.

## Introduction

Diabetes is one of the most common diseases, and its incidence has more than doubled in the past 20 years, making it an important public health issue^1^. Notably, diabetes and defective glucose intolerance increase cardiovascular disease risk by 3- to 8-fold^2^. Moreover, atherosclerosis is the primary cause of death in patients with diabetes with or without insulin resistance^3^. Therefore, there is an urgent need to unveil the precise mechanism by which diabetes accelerates atherosclerosis.

Accelerated atherosclerosis in diabetes involves lipid abnormalities, which lead to increased macrophage foam cell formation, a characteristic pathogenic event in atherosclerosis. Lipid accumulation interacts with oxidative stress, inflammation and insulin resistant in macrophages and promotes diabetic atherogenesis. Diabetic microenvironment signals, such as nutrient availability, oxidative stress, and inflammatory cytokines, influence macrophage metabolism, which in turn affects macrophage functionality. Accumulating data indicate that macrophages in specific microenvironments, such as inflammatory adipose tissues in obesity and diabetes, reprogram their metabolism to accomplish specific functions, e.g., cell survival, proliferation, phagocytosis, and inflammatory cytokine production^4,5^. On the other hand, macrophage metabolism governs function^6,7^. For example, excessive succinate production in pro-inflammatory macrophages stimulates hypoxia-inducible factor-1α expression, and then promotes interleukin 1β production, which aggravates the pro-inflammatory status^4^. Accordingly, there is great potential to modulate macrophage function by reprogramming metabolism, which would be beneficial to reduce diabetic atherogenesis promoted by macrophages^4–9^. Therefore, it is important to characterize the metabolic reprogramming and to identify potential therapeutic targets associated with lipid accumulation in macrophages, a characterized pathological event in diabetic atherosclerosis.

In this study, oleate, a prominent fatty acid in dietary and endogenous fatty acid, was used as a nutrient factor to induce lipid accumulation and relevant metabolic disturbances in macrophages. Resveratrol (RSV) is a natural plant polyphenol that is used to treat various metabolic diseases owing to its anti-inflammatory, anti-oxidative, anti-diabetic, and anti-atherosclerotic effects^10–13^. Metabolomics aims to comprehensively measure metabolic responses of living systems to pathophysiological or genetic stimuli in qualitative and quantitative manners^14^. Accordingly, an untargeted metabolomics approach based on gas chromatography–mass spectrometry (GC–MS) was first employed in this study to characterize the metabolic reprograming and to identify potential regulatory targets associated with lipid accumulation in macrophages, as well as to ascertain the protective effects of RSV. Furthermore, the effects of the potential regulatory targets related to lipid accumulation in macrophages were verified using specific agonists and inhibitors. To the best of our knowledge, this study is the first to demonstrate that peroxisome proliferator-activated receptor (PPAR) α and PPARγ activation alleviates total free fatty acid (FFA) and triglyceride (TG) accumulation in macrophages treated with oleate by repressing extracellular FFA import through the suppression of fatty acid transport protein 1 (FATP1*)* expression. Therapeutic strategies focused on activating PPAR and inhibiting FFA import and TG synthesis are promising approaches to reduce both diabetic and non-diabetic atherogenesis.

## Results

### Significant metabolic changes related to neutral lipid accumulation in macrophages

**Fig. 1:**
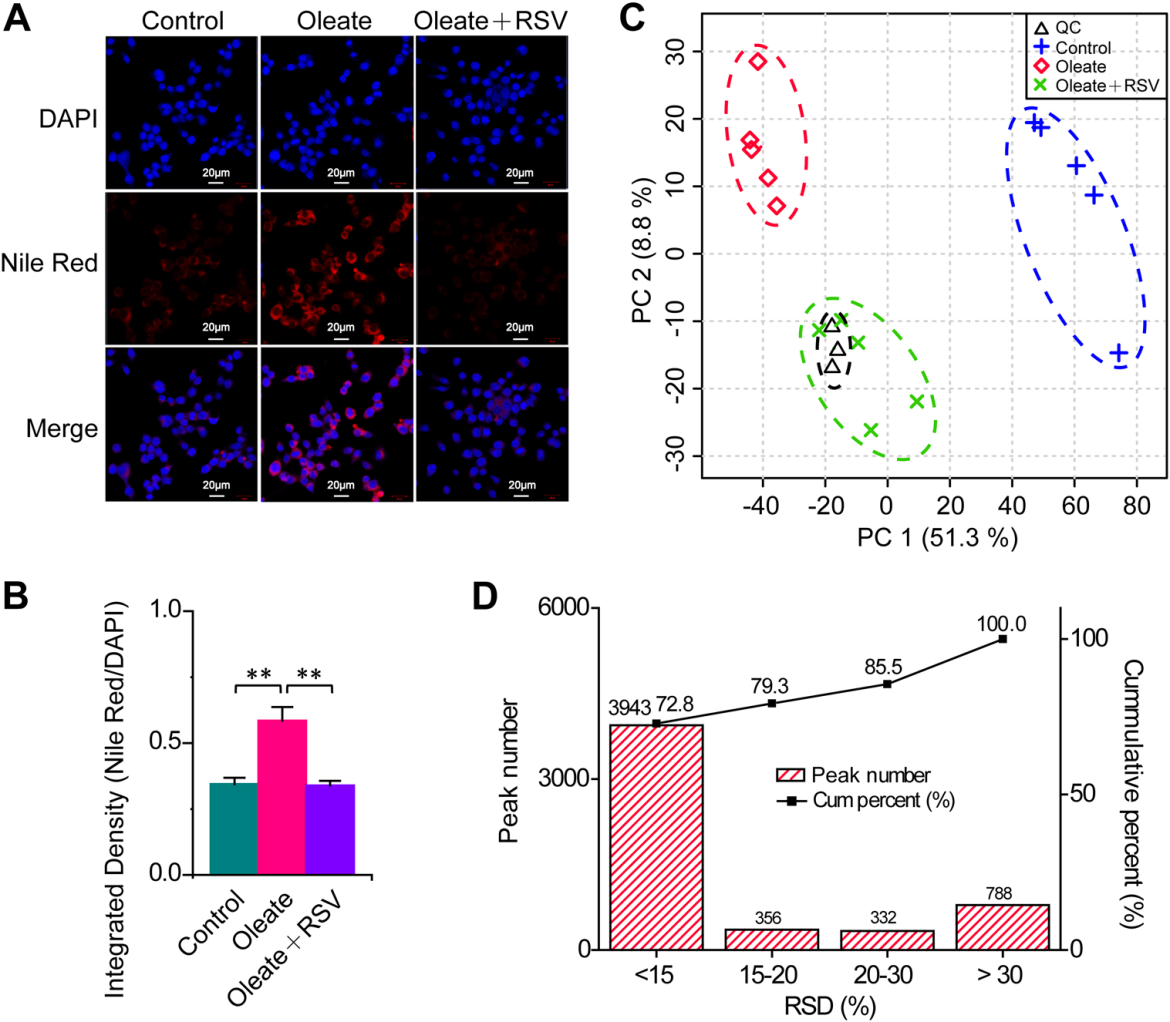
Significant changes in metabolic profiles related to neutral lipid accumulation in macrophages. **a** Representative Nile red staining of macrophages. **b** Quantification of lipid changes. Data were presented as the mean + SD. *n* = 8 per group. **P < 0.01, independent samples *t*-test. **c** Changes in metabolic profiles of macrophages treated with oleate and RSV. *n* = 5, 5, 5, and 3 in the control, oleate, oleate + RSV and QC (quality control) groups, respectively. **d** Relative standard deviation (RSD) distribution of ion peaks in 3 QC samples

Nile red staining of macrophages revealed that neutral lipids significantly accumulated in oleate-treated macrophages, and this accumulation was completely abolished by RSV (Fig. 1a, b). Accordingly, an untargeted metabolomics approach employing GC–MS was applied to obtain metabolic characteristics and identify key regulatory factors related to neutral lipid accumulation in macrophages. The 3 quality control (QC) samples were located close to each other in the principal component analysis score plot (Fig. 1c). Moreover, the relative standard deviation of the contents in 72.8, 79.3, and 85.5% of the 5419 ion peaks was less than 15, 20, and 30% in the QC samples, respectively (Fig. 1d). These data show that the metabolomics approach is repeatable and stable^15,16^.

**Fig. 2:**
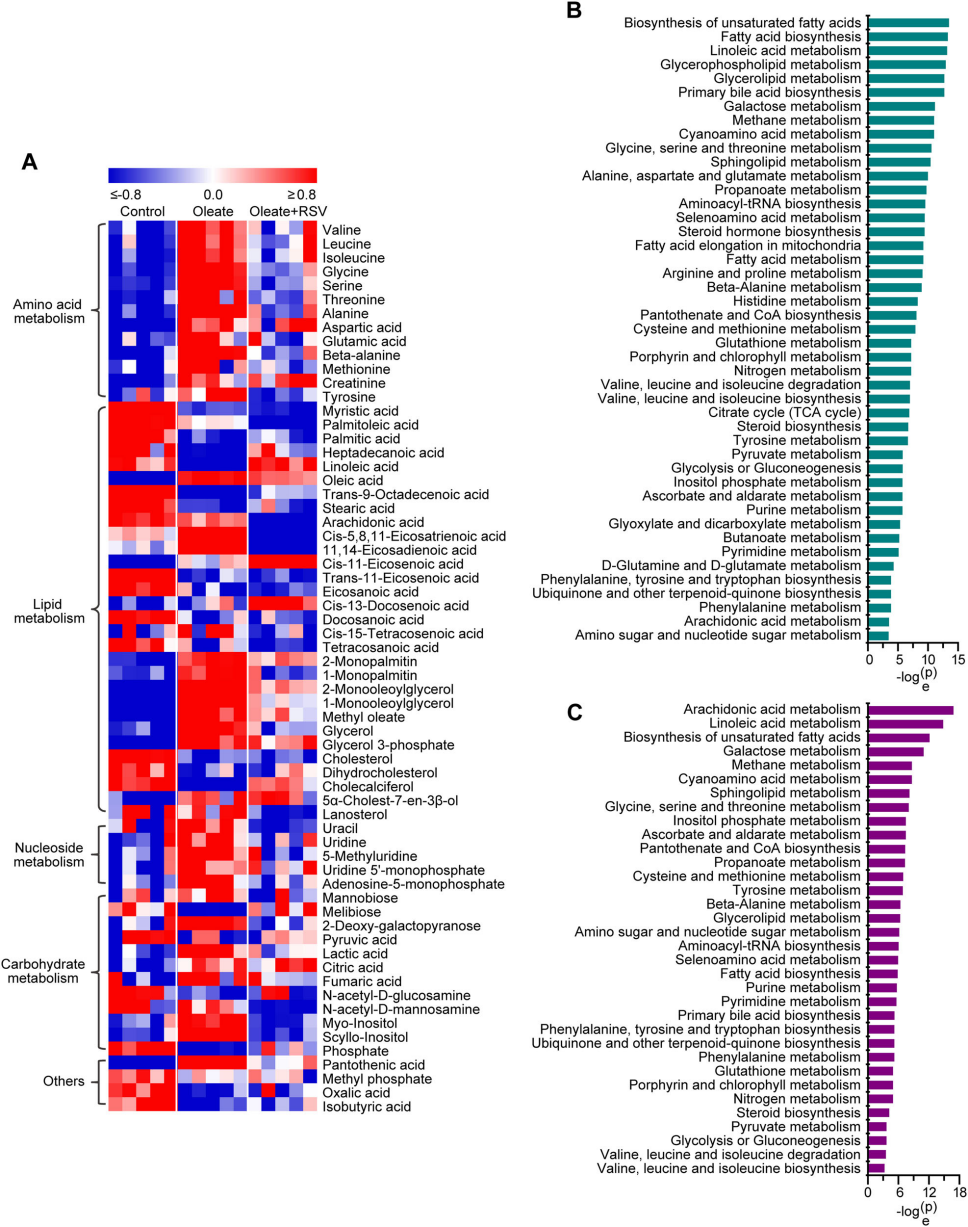
Significant metabolic changes related to neutral lipid accumulation in macrophages. **a** Heat map of metabolic changes in macrophages treated with oleate and RSV. After subtracting the mean, the levels of metabolites were divided by SD, and the data were then used to generate a heat map. **b** Pathway analysis of metabolic changes in macrophages treated with oleate. Significantly disturbed metabolic pathways are displayed (*P* < 0.05). **c** Pathway analysis of metabolic changes in macrophages treated with RSV. Significantly disturbed metabolic pathways are displayed (*P* < 0.05)

The metabolic profile of macrophages treated with oleate was significantly different from that of control macrophages in the principal component analysis score plot, and there was a substantial difference between the oleate and RSV treatment groups, indicating that prominent differences in metabolic profiles were induced during neutral lipid accumulation in macrophages and RSV treatment (Fig. 1c). Subsequently, 64 metabolites were identified mainly based on the retention time, retention indexes, and mass spectra (Fig. 2a). Forty-three of the 64 differential metabolites were verified by available standard references. The heat map indicated that metabolites involved in amino acid metabolism, lipid metabolism, nucleoside metabolism, carbohydrate metabolism, and other metabolic pathways were significantly disturbed in macrophages treated with oleate and RSV (Fig. 2a). In-depth pathway analysis revealed that 45 metabolic pathways were significantly perturbed during neutral lipid accumulation in macrophages, e.g., biosynthesis of unsaturated fatty acids; fatty acid biosynthesis; glycerolipid metabolism; glycine, serine and threonine metabolism; fatty acid metabolism; purine metabolism; and pyrimidine metabolism (Fig. 2b). In addition, 34 metabolic pathways were significantly disturbed in macrophages treated with oleate and RSV, including arachidonic acid metabolism; linoleic acid metabolism; glycine, serine and threonine metabolism; glycerolipid metabolism; fatty acid biosynthesis; purine metabolism; and pyrimidine metabolism (Fig. 2c).

Notably, most metabolites involved in amino acid metabolism and nucleoside metabolism and monoglycerides were significantly increased in oleate-treated macrophages, and these effects were significantly attenuated or abolished by RSV (Fig. 2a). Substantial changes in fatty acids were also observed during neutral lipid accumulation in macrophages, and most of the changes were attenuated by RSV (Fig. 2a). These data suggest potential roles of amino acids, nucleosides, FFAs and monoglycerides in neutral lipid accumulation in macrophages. The detailed metabolic changes during lipid accumulation in macrophages are provided below.

### Significant changes in lipid metabolism related to neutral lipid accumulation in macrophages

**Fig. 3:**
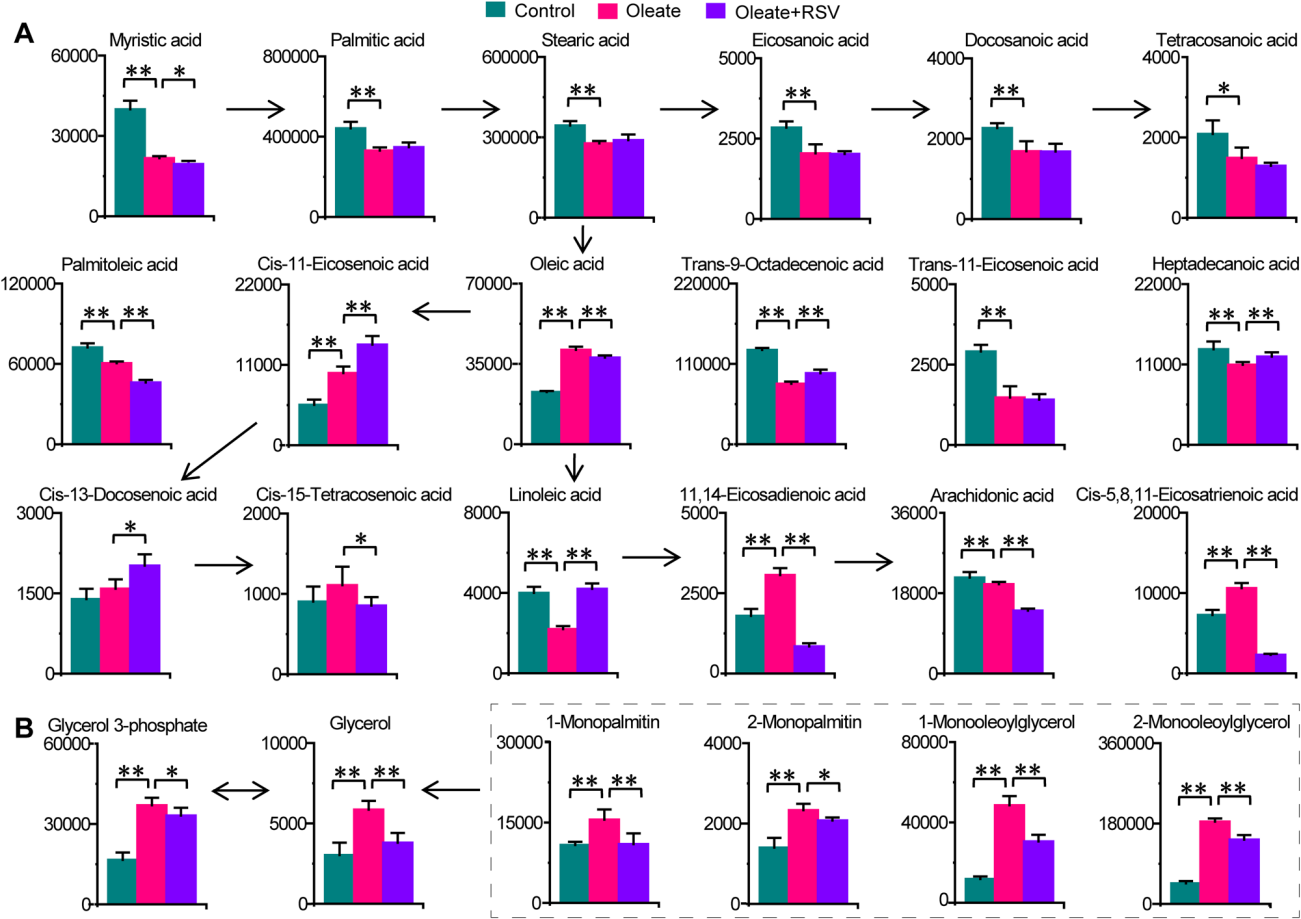
Significant changes in lipid metabolism related to neutral lipid accumulation in macrophages. Columns represent the mean + SD. *P < 0.05, **P < 0.01, two-tailed Mann–Whitney *U*-test. **a** FFA changes in macrophages treated with oleate and RSV. **b** Monoglyceride changes in macrophages treated with oleate and RSV

Significant changes in FFAs and monoglycerides were induced during neutral lipid accumulation and RSV treatment (Fig. 3). Most FFAs, such as palmitic acid, palmitoleic acid, stearic acid, linoleic acid, and eicosanoic acid, were all significantly decreased in oleate-treated macrophages. In contrast, oleic acid, 11,14-eicosadienoic acid and 5,8,11-eicosatrienoic acid were significantly increased in oleate-treated macrophages, and these effects were attenuated or abolished by RSV. Notably, glycerol, glycerol 3-phosphate, 1-monopalmitin, 2-monopalmitin, 1-monooleoylglycerol, and 2-monooleoylglycerol significantly accumulated in oleate-treated macrophages, but these accumulation events were alleviated by RSV. These data indicate the role of FFAs and monoglycerides in neutral lipid accumulation in macrophages and RSV treatment.

### Significant changes in amino acid metabolism, the tricarboxylic acid cycle, glycolysis/gluconeogenesis, nucleoside metabolism, and carbohydrate metabolism related to neutral lipid accumulation in macrophages

**Fig. 4:**
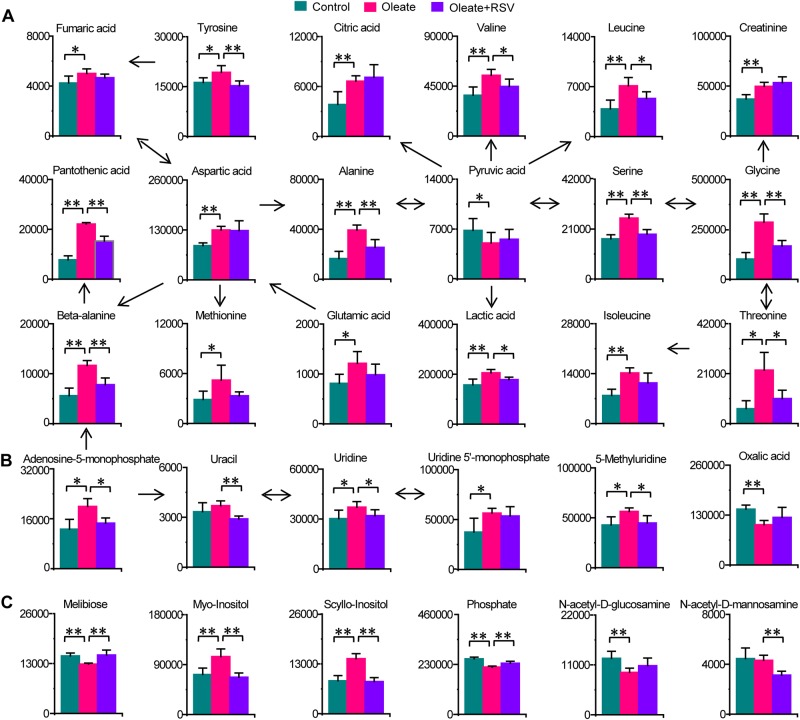
Significant changes in amino acid metabolism, glycolysis/gluconeogenesis, the tricarboxylic acid cycle, nucleoside metabolism, and carbohydrate metabolism related to neutral lipid accumulation in macrophages. Columns represent the mean + SD. *P < 0.05, **P < 0.01, two-tailed Mann–Whitney *U*-test. **a** Changes in amino acid metabolism, the tricarboxylic acid cycle, and glycolysis/gluconeogenesis in macrophages treated with oleate and RSV. **b** Changes in nucleoside metabolism in macrophages treated with oleate and RSV. **c** Changes in carbohydrate metabolism in macrophages treated with oleate and RSV

Significant changes in metabolites involved in amino acid metabolism were also observed during neutral lipid accumulation in macrophages and upon RSV treatment (Fig. 4a). Alanine, aspartate, glutamate, glycine, serine, threonine, valine, isoleucine, beta-alanine, pantothenic acid, tyrosine, and methionine were significantly increased in oleate-treated macrophages, and most of the increases were significantly attenuated by RSV. Lactate, fumarate and citrate were significantly increased, while pyruvate was significantly reduced in oleate-treated macrophages, and the increase in lactate was attenuated by RSV (Fig. 4a). In addition, adenosine-5-monophosphate, uridine and 5-methyluridine were significantly increased in oleate-treated macrophages, and these effects were significantly alleviated by RSV (Fig. 4b). Moreover, myo-inositol and scyllo-inositol were significantly increased, while melibiose and phosphate were significantly decreased in oleate-treated macrophages, and these effects were attenuated by RSV (Fig. 4c). These data suggest potential roles for amino acid metabolism (such as glycine, serine, and threonine metabolism; beta-alanine metabolism; and valine, leucine and isoleucine metabolism), glycolysis/gluconeogenesis, nucleoside metabolism, and carbohydrate metabolism (such as inositol phosphate metabolism) in neutral lipid accumulation in macrophages and RSV treatment.

### Oleate induces total FFA and TG accumulation and significant changes in relevant lipid signaling in macrophages

**Fig. 5:**
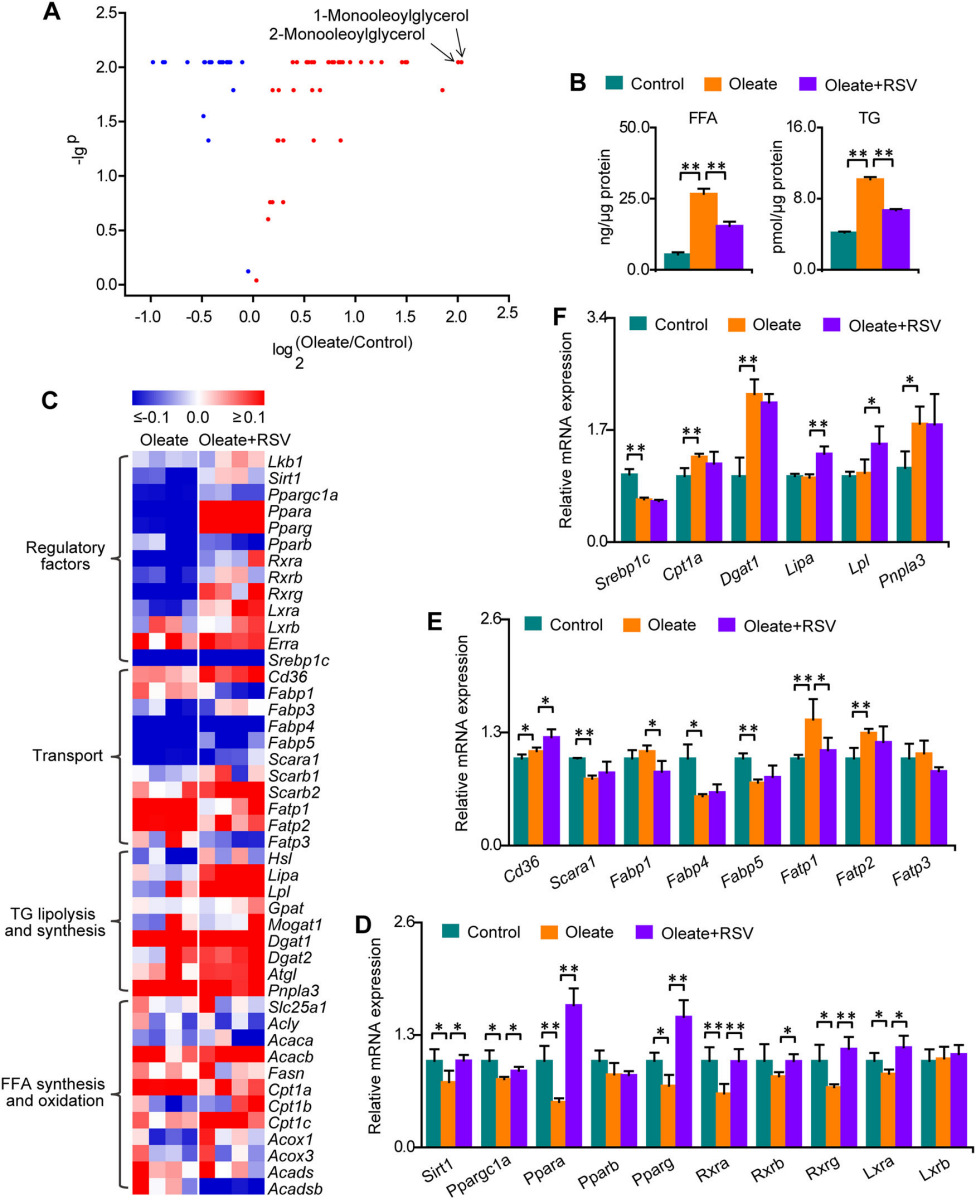
Oleate induces total FFA and TG accumulation and significant changes in relevant lipid signaling. Columns represent the mean + SD. *P < 0.05, **P < 0.01, independent samples *t*-test. **a** Volcano plot of metabolic changes in macrophages treated with oleate. *n* = 5 per group. **b** Changes in FFAs and TGs in macrophages treated with oleate. *n* = 3 per group. **c** Heat map of changes in the mRNA expression of genes related to TG metabolism in macrophages treated with oleate and RSV. The mRNA expression levels of genes in the oleate and oleate + RSV groups were divided by the average mRNA expression in the control group, and the base 10 logarithm was taken. *n* = 4 per group. **d** Changes in the mRNA expression of genes related to PPAR signaling. *n* = 4 per group. **e** Changes in the mRNA expression of genes related to FFA and TG transport. *n* = 4 per group. **f** Changes in the mRNA expression of genes related to the synthesis, lipolysis, and oxidation of FFAs and TGs. *n* = 4 per group

The volcano plot illustrates that 1-monooleoylglycerol and 2-monooleoylglycerol were the most altered metabolites during neutral lipid accumulation in macrophages (Fig. 5a). Monoglycerides can be further used for diglyceride and then TG synthesis, thus contributing to neutral lipid accumulation in macrophages. Moreover, FFAs participate in the synthesis and lipolysis of monoglycerides, diglycerides, and TGs. Therefore, we hypothesize that oleate induces disturbances in glycerolipid metabolism and then leads to TG accumulation in macrophages. As FFAs are separated and then detected by GC–MS, and the levels of FFAs differ greatly from each other, it is not suitable to obtain the total FFAs by directly summing the contents of all FFAs. Accordingly, total FFAs and TGs were measured using quantification assay kits. As expected, total FFAs and TGs were significantly increased in macrophages treated with oleate, and these effects were significantly attenuated by RSV (Fig. 5b).

Given that PPARs are ligand-activated nuclear receptors that control FFA and TG homeostasis, FFAs and their products are natural ligands for PPARs^17–20^. To test whether total FFA and TG accumulation in macrophages is regulated by PPARs and to determine how this regulation occurs, the mRNA expression levels of genes involved in PPAR signaling and of PPAR target genes related to FFA and TG homeostasis were measured. The heat map showed significant changes in the mRNA expression levels of genes related to PPAR signaling and the transport, synthesis, lipolysis and oxidation of FFAs and TGs (Fig. 5c). The mRNA expression levels of lipid regulatory factors related to PPARs, such as *Sirt1*, *Ppargc1a*, *Ppara*, *Rxra*, *Pparg*, *Rxrg*, and *Lxra*, were significantly decreased in oleate-treated macrophages, and all these effects were entirely abolished by RSV (Fig. 5d). However, significant alterations in *Pparb* and *Rxrb* mRNA expression were not observed in macrophages treated with oleate, and RSV could not effectively abolish the decrease in *Pparb* mRNA expression induced by oleate (Fig. 5d). These data suggest the potential protective effects of PPARα and PPARγ against total FFA and TG accumulation in macrophages treated with oleate.

**Fig. 6:**
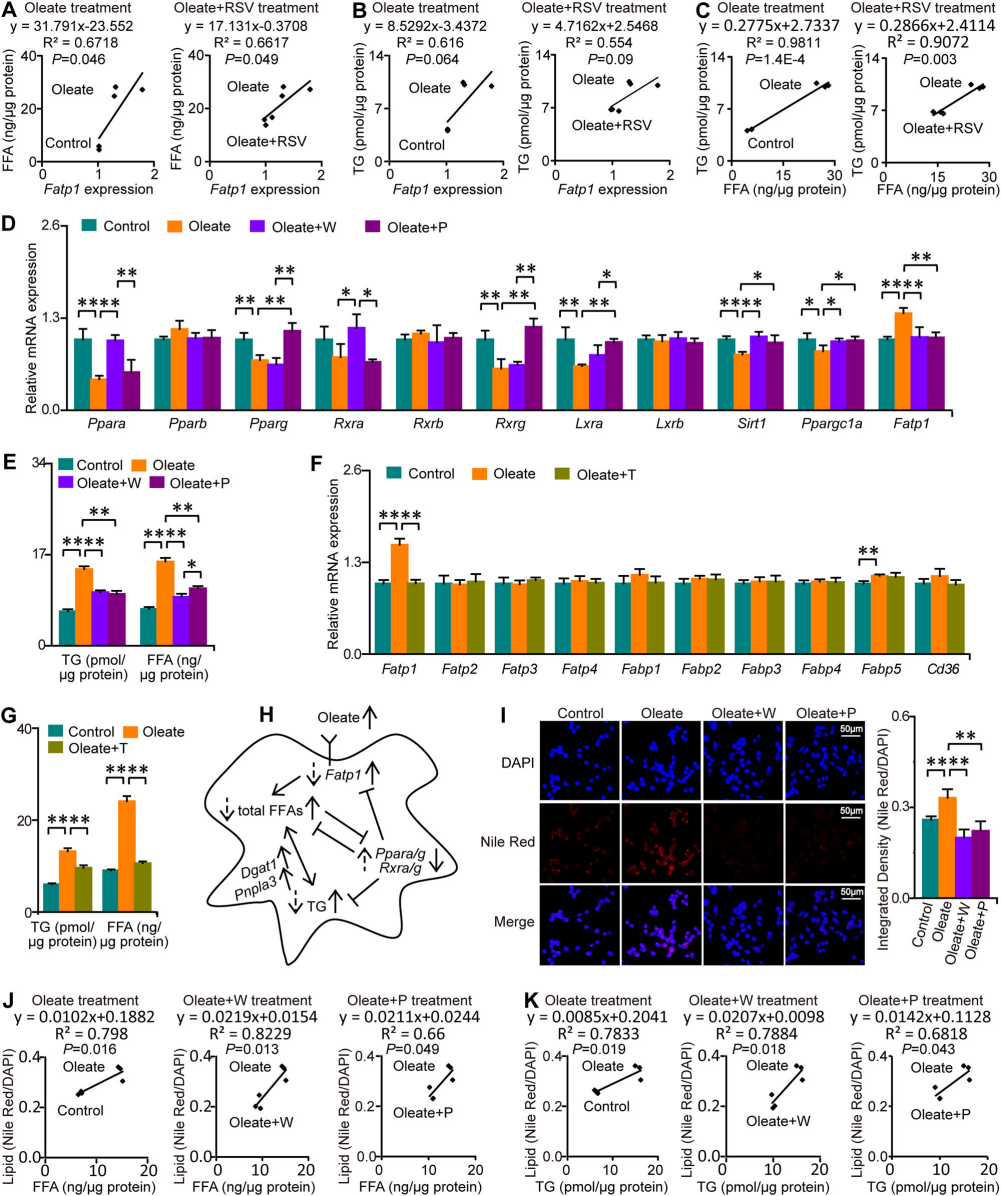
PPARα and PPARγ activation attenuates total FFA and TG accumulation in macrophages. Columns represent the mean + SD. *P < 0.05, **P < 0.01, independent samples *t*-test. W WY14643, P pioglitazone, T TNFα. **a** Associations between the levels of *Fatp1* expression and total FFAs. *n* = 3 per group. **b** Associations between the levels of *Fatp1* expression and TGs. *n* = 3 per group. **c** Associations between total FFA and TG content. *n* = 3 per group. **d** Selective PPARα and PPARγ agonists abolished the oleate-induced suppression of PPAR signaling. *n* = 4 per group. **e** Selective PPARα and PPARγ agonists attenuated oleate-induced FFA and TG accumulation. *n* = 3 per group. **f** Selective inhibition of FATP1 by TNFα. *n* = 4 per group. **g** Selective inhibition of FATP1 attenuated oleate-induced FFA and TG accumulation. *n* = 3 per group. **h** Molecular mechanisms by which PPARα and PPARγ attenuate oleate-induced FFA and TG accumulation in macrophages. Solid arrows on the right of terms: upward/downward arrows denote increases/decreases in response to oleate. Dotted arrows on the left of terms: upward/downward arrows denote increases/decreases in response to PPARα and PPARγ agonists. **i** Selective PPARα and PPARγ agonists abolished oleate-induced neutral lipid accumulation. *n* = 6–10 per group. **j** Associations between changes in FFAs and changes in neutral lipids in macrophages treated with oleate, oleate+W and oleate+P (*n* = 3 per group). **k** Associations between changes in TGs and changes in neutral lipids in macrophages treated with oleate, oleate+W and oleate+P (*n* = 3 per group)

The mRNA expression levels of *Cd36*, *Fatp1*, *Fatp2*, *Dgat1*, *Cpt1a*, and *Pnpla3* were significantly upregulated, while those of *Scara1*, *Fabp4*, *Fabp5*, and *Srebp1c* were significantly downregulated in oleate-treated macrophages (Fig. 5e, f). These data indicate disordered lipid transport, increased FFA import into mitochondria for oxidation, and enhanced TG synthesis and lipolysis during neutral lipid accumulation in oleate-treated macrophages. On the other hand, the mRNA expression levels of *Cd36*, *Lipa*, and *Lpl* were significantly increased and those of *Fabp1* and *Fatp1* were significantly decreased in macrophages cotreated with oleate and RSV (Fig. 5e, f). Notably, the significant increase in *Fatp1* mRNA expression during neutral lipid accumulation in macrophages was markedly alleviated by RSV. Pearson correlation analysis revealed a significant correlation between *Fatp1* mRNA expression and total FFA level during neutral lipid accumulation and RSV treatment, suggesting that total FFA accumulation in macrophages treated with oleate was probably due, at least in part, to accelerated FFA import into the cell via FATP1, and this effect was attenuated by RSV (Fig. 6a). Although a significant correlation between *Fatp1* mRNA expression and TG level was not observed, the level of total FFAs was highly correlated with that of TGs (*R*^2^ > 0.9), which indicated that TG accumulation in oleate-treated macrophages was probably due to total FFA accumulation, and this effect was attenuated by RSV (Fig. 6b, c). Taken together, oleate-induced total FFA and TG accumulation is probably due to enhanced FFA import via FATP1, which is regulated by PPARα and PPARγ signaling.

### PPARα and PPARγ activation attenuates total FFA and TG accumulation in macrophages via the suppression of *Fatp1* expression

To confirm the role of PPARα and PPARγ in total FFA and TG accumulation in macrophages treated with oleate, these cells were cotreated with selective agonists of PPARα (WY14643) or PPARγ (pioglitazone) at 0.1 μg/ml to form the oleate + WY14643 and oleate + pioglitazone groups, and the mRNA expression of *Fatp1* and other genes related to PPAR signaling was measured (Fig. 6d). As expected, the marked reduction in *Ppara* and *Rxra* mRNA expression in oleate-treated macrophages was completely eliminated by WY14643, but not by pioglitazone. Meanwhile, the significant decrease in *Pparg* and *Rxrg* mRNA expression in oleate-treated macrophages was entirely abolished by pioglitazone but not by WY14643. However, significant alterations in *Pparb* and *Rxrb* mRNA expression were not observed in macrophages treated with oleate and the agonists. Moreover, the marked decrease in *Sirt1*, *Ppargc1a*, and *Lxra* mRNA expression and increase in *Fatp1* mRNA expression were completely abolished by both WY14643 and pioglitazone. Furthermore, we verified that total FFA and TG accumulation in macrophages treated with oleate was significantly alleviated by both WY14643 and pioglitazone (Fig. 6e). These data demonstrate the high selectivity of WY14643 and pioglitazone for PPARα and PPARγ, respectively, and the regulatory role of PPARα and PPARγ signaling in total FFA and TG accumulation in macrophages treated with oleate.

To further determine the role of FATP1 in total FFA and TG accumulation in oleate-treated macrophages, macrophages were cotreated with tumor necrosis factor α (TNFα) to suppress the expression of FATP1. As expected, TNFα treatment abolished the increase in the mRNA expression level of *Fatp1* in oleate-treated macrophages, but had no significant effects on the mRNA expression levels of other FFA transport proteins, including *Fatp2-4*, *Fabp1-5*, and *Cd36* (Fig. 6f). Furthermore, oleate-induced accumulation of total FFAs and TGs in macrophages were significantly alleviated after TNFα treatment (Fig. 6g). These data demonstrated the high specificity of TNFα for the inhibition of FATP1, and the regulatory role of FATP1 in FFA import and consequent accumulation of total FFAs and TGs in oleate-treated macrophages.

Excessive FFAs imported via FATP1 can be stored in the form of TGs synthesized from diacylglycerol O-acyltransferase 1 and can also be released from TGs via lipolysis in macrophages treated with oleate. The fold change in *Dgat1* mRNA expression was greater than that in *Pnpla3* mRNA expression, which indicated a larger role of synthesis than of lipolysis, thus leading to TG accumulation in macrophages treated with oleate. On the other hand, PPARα and PPARγ activation by selective agonists inhibited extracellular FFA import via FATP1, thus alleviating total FFA and TG accumulation in oleate-treated macrophages (Fig. 6h).

We also found that total neutral lipid accumulation in oleate-treated macrophages was completely abolished by both WY14643 and pioglitazone, which indicated that neutral lipid accumulation in such macrophages was mediated by PPARα and PPARγ (Fig. 6i). In addition, total FFA or TG levels were significantly correlated with neutral lipid levels in macrophages treated with oleate, oleate + WY14643 or oleate + pioglitazone, suggesting that total FFA and TG accumulation contributed to neutral lipid accumulation in oleate-treated macrophages (Fig. 6j, k). Therefore, PPARα and PPARγ activation is an effective strategy for reducing total FFA, TG, and neutral lipid accumulation in macrophages, which is beneficial for reducing the risk of diabetic atherosclerosis.

## Discussion

Lipid accumulation in macrophages, a vital pathological event in atherogenesis, interacts with microenvironment signals and then accelerates diabetic atherosclerosis. Diabetic microenvironment signals shape macrophage metabolism, which in turn governs macrophage function. Hence, there is the potential to modulate macrophage function by reprogramming their metabolism, which would be beneficial for alleviating the risk of diabetic atherosclerosis characterized by the formation of macrophage foam cells. Accordingly, an unbiased metabolomics approach was undertaken in this study to characterize the metabolic signatures and to identify potential regulatory targets related to lipid accumulation in oleate-treated macrophages.

We discovered that amino acid metabolism (e.g., alanine, aspartate, and glutamate metabolism; glycine, serine, and threonine metabolism; beta-alanine metabolism; valine, leucine, and isoleucine metabolism; and tyrosine metabolism) was associated with lipid accumulation in macrophages treated with oleate and RSV. Aside from being utilized for protein synthesis, amino acids can be utilized for energy production, and the synthesis of lipids, and nucleosides to support cell growth and proliferation. In this study, PPARα and PPARγ signaling was inhibited in oleate-treated macrophages, which stimulated numerous genes involved in amino acid metabolism, including transamination, deamination, inter-conversions of amino acids, and oxidation of α-keto acids^21^. The increased levels of glutamate, aspartate and alanine in macrophages treated with oleate could be ascribed to the activation of glutaminase, glutamate dehydrogenase, glutamic-oxaloacetic transaminase, alanine-glyoxylate aminotransferase, and asparagine synthetase owing to the inhibition of PPARα and PPARγ signaling^21^. Accelerated transamination and deamination implicated in alanine, aspartate, and glutamate metabolism produced more oxaloacetate for citrate synthesis, and more aspartate and glutamate for de novo nucleoside synthesis in macrophages treated with oleate, which was verified by the increased levels of citrate and nucleosides. The above metabolic processes were alleviated, or even abolished, through the activation of PPAR signaling by RSV. In addition, the inhibition of PPARα and PPARγ signaling in macrophages by oleate activated the oxidation of α-keto acids via branched-chain keto-acid dehydrogenase, which accelerates the degradation of branched-chain amino acids to replenish tricarboxylic acid cycle intermediates via acetyl-CoA and/or succinyl-CoA, and promoted citrate synthesis^21^. Moreover, the suppression of PPARα and PPARγ signaling in oleate-treated macrophages stimulated alanine-glyoxylate aminotransferase, phenylalanine hydroxylase, and argininosuccinate lyase, leading to the accumulation of glycine, serine, threonine, tyrosine, and fumarate in this study^21^. The increased levels of glycine, serine, and threonine in macrophages treated with oleate represented more precursors for de novo nucleoside synthesis and methylation^22^.

Notably, we observed substantial increases in the levels of total FFAs and TGs in macrophages treated with oleate, and these effects were significantly alleviated by RSV and PPARα and PPARγ agonists. Total FFAs accumulated in oleate-treated macrophages with the increased *Fatp1* expression, and this effect was significantly ameliorated by RSV and PPARα and PPARγ agonists. Excessive FFAs were stored in the form of TGs, and this effect was attenuated by RSV and PPARα and PPARγ agonists through the suppression of FFA uptake via FATP1. FFA handling and homeostasis are perturbed in obesity and diabetes, leading to impaired FFA clearance and storage and resulting in FFA accumulation in the circulation and various tissues, such as liver, skeletal muscle, and arterial tissues in the form of TGs, diacylglycerols, cholesteryl ester, ceramides, or fatty acyl-CoA, thereby accelerating lipotoxic effects, insulin resistance, and atherosclerosis^23^. FATP1, an integral membrane protein with long-chain and very long-chain fatty acyl-CoA synthetase activity, has vital roles in facilitating FFA influx, therefore affecting TG-rich lipid droplet size and other lipid pools in mammalian cells^23–26^. FFAs imported by FATP1 are transformed into acyl-CoA derivatives and preferentially utilized for TG synthesis^26,27^. FATP1 overexpression increases long-chain FFA influx and TG accumulation, while FATP1 knockdown decreases long-chain FFA import coupled with decreased levels of acyl-CoA, monoacylglycerols, diacylglycerols, and TGs^26^. Moreover, transgenic expression of FATP1 in the heart induces a four-fold increase in myocardial FFA uptake, which contributes to early cardiomyocyte FFA accumulation, impaired left ventricular filing, biatrial enlargement, and diastolic dysfunction in mice^28^. Furthermore, FATP1 ablation protects mice from the impaired glucose uptake, glycogen synthesis, and glycolysis that occurs in wild-type mice treated with a high-fat diet or a lipid infusion^23^.

PPAR-RXR (retinoid X receptor) transcriptional complexes have vital roles in energy homeostasis, including the handling and storage of FFAs and TGs, and in glucose homeostasis, which is highly correlated with obesity, diabetes, and atherosclerosis^29^. FFAs and their derivatives are natural ligands for PPAR and have different effects on transcriptional regulation by PPARs, which involves heterodimerization with RXR, and coregulator recruitment and release^18,30^. The treatment of macrophages with oleate induced conformational changes in the ligand-binding domain of PPAR, which inhibited the expression of *Ppara*, *Pparg*, *Rxra*, and *Rxrg*, resulting in decreased formation of the PPARα-RXRα and PPARγ-RXRγ transcriptional complexes. Moreover, *Ppargc1a* expression was suppressed in macrophages treated with oleate, which led to decreased recruitment of peroxisome proliferator-activated receptor gamma coactivator 1-α by PPARα or PPARγ, further affecting the transcriptional regulation of target genes by the PPAR-RXR transcriptional complex. Subsequently, the expression of *Fatp1*, a target gene of PPARα and PPARγ, was activated, leading to increased FFA influx and the subsequent total FFA and TG accumulation in oleate-treated macrophages. As expected, the effects of oleate on total FFA and TG accumulation in macrophages were attenuated by activating PPARα or PPARγ signaling through RSV and a specific agonist. Furthermore, PPARα and PPARγ activation eliminated neutral lipid accumulation in oleate-treated macrophages.

Taken together, the results show that most amino acids, nucleosides, lactate, monoacylglycerols, total FFAs and TGs accumulated during lipid accumulation in oleate-treated macrophages, and these accumulation events were effectively attenuated or even abolished by RSV. Notably, 1-monooleoylglycerol and 2-monooleoylglycerol showed the largest fold change in accumulations. Meanwhile, PPARα and PPARγ signaling was inhibited in macrophages treated with oleate, but this effect was abolished by RSV. Furthermore, we discovered that oleate induced total FFA and TG accumulation by promoting FFA import via the activation of *Fatp1* expression, and this effect was attenuated by activating PPARα or PPARγ signaling with RSV and a specific agonist. To our knowledge, this study provides the first evidence that accumulation of amino acids, nucleosides, lactate, monoacylglycerols, total FFAs, and TGs in oleate-treated macrophages is effectively attenuated or even abolished by RSV, and that the activation of PPARα and PPARγ attenuates total FFA and TG accumulation by inhibiting FFA influx through the suppression of *Fatp1* expression in macrophages treated with oleate. The results of this study suggest that therapeutic strategies aims at reducing total FFA and TG accumulation by activating PPAR signaling, repressing FFA import and TG synthesis, and promoting FFA oxidation are promising approaches to reduce the risk of obesity, diabetes and atherosclerosis. However, the effects of PPARs and FATP1 are influenced by specific ligands, cells, tissues, organs, and physiological status, and that negative effects of PPARs, such as the upregulation of CD36 by PPAR activation, which promotes FFA uptake, should not be ignored.

## Materials and methods

### Materials

DMSO (≥99.7%), resveratrol (99.0%), oleic acid (99.0%), pioglitazone, WY-14643, TNFα, pyridine (99.8%), methoxyamine hydrochloride (98%), and N-methyl-N-(trimethylsilyl)-trifluoroacetamide (≥98.5%) were obtained from Sigma-Aldrich (Shanghai, China). RAW264.7 macrophages were purchased from Cell Bank of Chinese Academy of Science (Shanghai, China). DMEM (high glucose) and primers were obtained from Shanghai Sangon Biotech (Shanghai, China) and HyClone (USA), respectively.

### Cell culture and treatments

RAW264.7 cells were cultured in high-glucose DMEM medium (HyClone) supplemented with 10% fetal bovine serum in a humidified atmosphere with 5% CO_2_ at 37 °C. Macrophages were treated with 1‰ DMSO (control), 65 μg/ml oleate or 65 μg/ml oleate plus 1.5 μg/ml RSV for 24 h to identify metabolic signatures and potential regulatory factors related to lipid accumulation in RAW264.7 cells. Furthermore, RAW264.7 cells were treated with 1‰ DMSO (control), oleate (65 μg/ml), oleate (65 μg/ml) plus WY-14643 (1.5 μg/ml), oleate (65 μg/ml) plus pioglitazone (1.5 μg/ml) or oleate (65 μg/ml) plus TNFα (1 μg/ml) for 24 h to determine the role of PPARs and FATP1 in total FFA, TG, and neutral lipid accumulation.

### Nile red staining

Macrophages were fixed in 4% paraformaldehyde for 40 min and then washed three times with PBS. After being stained with DAPI (2.5 μg/ml in methanol) at 37 °C for 15 min, the macrophages were washed twice with methanol. Subsequently, the macrophages were stained with Nile red (10 μg/ml in methanol) at 37 °C for 0.5 h and then washed with PBS. Confocal fluorescence imaging of cells was performed employing a confocal microscope (LSM 7100, Zeiss, Germany). The integrated density ratio (Nile Red/DAPI) was determined to quantify the cellular lipid content.

### Cell sample preparation for metabolomics analysis

After culture and treatment, macrophages were washed with cold PBS and collected using scrapers. Following centrifugation at 5000 rpm for 5 min, the cells were frozen in liquid nitrogen and then stored at −80 °C for subsequent sample preparation prior to analysis. Then, 1000 μL of ice-cold methanol/water (v/v = 4:1) was added to the cell sample, which was vortexed for 1 min. Subsequently, the cells were centrifuged at 13,000 rpm for 15 min at 4 °C. Eight hundred microliters of the supernatant was pipetted into a centrifuge tube and dried in a SpeedVac concentrator (Thermo Scientific, USA). The dried sample was resuspended in 50 μL of methoxyamine hydrochloride (20 mg/mL in pyridine), and the mixture was vortexed for 30 s and then placed in a water bath for 1.5 h at 37 °C for the oximation reaction. Then, 40 μL of N-methyl-N-(trimethylsilyl)-trifluoroacetamide was added to the sample, which was vortexed for 10 s and then placed in a water bath for 1.0 h at 37 °C for the silylation reaction. Finally, the derivatized sample was centrifuged at 13,000 rpm for 15 min at 4 °C, and the supernatant was injected for subsequent analysis. To evaluate the stability and repeatability of the metabolomics approach, QC samples were prepared by mixing the remaining supernatant from all samples and dividing into 800-μL aliquots. One QC sample was inserted every 5 analytical samples and processed in the same way as the other samples regarding vacuum drying, derivatization, analysis, and data processing.

### Instrumental metabolomics analysis

Metabolic profiles of the samples were obtained using a GC–MS system (GCMS-QP 2010 plus, Shimadzu, Japan) equipped with an AOC-20i autosampler. Instrumental parameters were similar to those applied in our previous studies^16,31,32^. One microliter of the sample was injected. A DB-5 MS capillary column (30 m × 250 μm × 0.25 μm, J&W Scientific Inc., USA) was employed for metabolite separation. The split ratio and constant linear velocity of helium (carrier gas) were set to 2:1 and 40.0 cm/s, respectively. The oven temperature was maintained at 70 °C for 3.0 min, increased to 300 °C at a rate of 5 °C/min, and then maintained at 300 °C for 10 min. The temperatures of the inlet, interface and ion source were maintained at 300, 280, and 230 °C, respectively. Electron impact (70 eV) was employed as the ionization mode. The detector voltage was set according to the tuning results. Mass signals (m/z, 33–600) were acquired using GCMS solution 2.7 (Shimadzu, Japan) in full scan mode. The solvent delay time and event time were 5.5 min and 0.2 s, respectively. Finally, a light diesel sample was analyzed under the same parameters as the analytical samples to obtain the retention time of n-alkanes, which was required for calculating the retention indexes of the metabolites.

### Data preprocessing for metabolomics analysis

Raw mass data in NetCDF format generated by GCMS solution 4.2 (Shimadzu, Japan) were utilized for peak matching using XCMS^33^. Feature ions of metabolites were generated via the deconvolution of mass signals using ChromaTOF 4.43 (LECO Corporation, USA). Metabolites were identified mainly according to automatic and manual spectral comparisons and confirmed by available reference standards based on the retention time, retention indexes and mass spectra. Ion peaks of metabolites were divided by total ion current and multiplied by 1 × 10^8^, and the data were then subjected to statistical analysis.

### RNA extraction and RT-PCR

Total RNA was extracted from macrophages using TRIzol reagent (Thermo Fisher Scientific, MA, USA). PrimeScript™ RT master mix (Takara, Dalian, China) was used to reverse transcribe RNA to cDNA. Real-time RT-PCR was performed with SYBR® Premix Ex Taq™ _II_ (Takara, Dalian, China). β-actin was employed as the internal standard to normalize gene expression via the 2^–ΔΔCt^ method. Primers for Q-PCR analysis were designed using the NCBI database (Supplementary Table 1).

### Determination of total FFAs and TGs

Macrophages were washed with cold PBS and then collected using scrapers. Eighty percent of the collected cells were centrifuged at 2500 rpm for 5 min at 4 °C, and intracellular total FFA levels in the pellets were measured using a Free Fatty Acid Quantification Assay Kit (Abcam) based on the manufacturer’s instructions. Twenty percent of the collected cells were used to determine the protein level, which was applied to normalize the total FFA content. Similar to the total FFA determination, 80% of the collected cells were used to detect intracellular TG levels using a Triglyceride Quantification Assay Kit (Abcam) according to the instructions, and the protein level in 20% of the collected cells was used for normalization.

### Statistical analysis

Principal component analysis and pathway analysis were performed using MetaboAnalyst 3.0^34^. A non-parametric test (two-tailed Mann–Whitney *U*-test) and independent sample *t*-tests were performed by MeV 4.9.0 and PASW Statistics 18 (SPSS Inc., Chicago, USA), respectively, to evaluate differences in metabolite content, mRNA expression and fluorescence intensity among groups^35^. The Pearson correlation coefficient was employed to evaluate bivariate correlations among levels of *Fatp1*, FFAs, TGs, and neutral lipids using PASW Statistics 18. The significance level was 0.05. The heat map was generated via MeV 4.9.0.

## Electronic supplementary material

Supplementary Table 1 Primers for RT-PCR analysis
