# Supplementary material for: PPARα and PPARγ activation attenuates total free fatty acid and triglyceride accumulation in macrophages via the inhibition of Fatp1 expression
Source: Cell Death Dis. 2019 Jan 15;10(2):39. doi: 10.1038/s41419-018-1135-3 (PMC6426939; doi:10.1038/s41419-018-1135-3)
Supplement: Supplementary file 1 — Supplementary Table 1 Primers for RT-PCR analysis [file 41419_2018_1135_MOESM1_ESM.docx]

**Supplementary Table 1 Primers for RT-PCR analysis.**

| **Primers** | **Primer Sequence** |
| --- | --- |
| *Lkb1* | F: ACGGCCTGGAATACCTACAC  R: CCATTGGTGGTGAGTAGCAG |
| *Sirt1* | F: GAGCTGGGGTTTCTGTCTCC  R: CCGCAAGGCGAGCATAGATA |
| *Ppargc1a* | F: GTGTTCTGGTACCCAAGGCA  R: ATGGTCACCAAACAGCCGAA |
| *Ppara* | F: CTTCCCAAAGCTCCTTCAAAAA  R: CTGCGCATGCTCCGTG |
| *Pparb* | F: GATGACAGTGACCTGGCGCT  R: AGGCCTGGCCGGTCTC |
| *Pparg* | F: GACAGGAAAGACAACGGACAAA  R: GCTTCTACGGATCGAAACTGG |
| *Rxra* | F: TGTGGATCTTTGGGGTGCAG  R: TGAGTAAAGATGGCGAGAGTGG |
| *Rxrb* | F: CCTGACCTACTCGTGTCGTG  R: GCGACAGTACTGACAGCGAT |
| *Rxrg* | F: GAATGAACTGAGCAGCCCAAC  R: CAAGGCTACTGAAGGGCTCA |
| *Lxra* | F: CTGAAGCGGCAAGAAGAGGA  R: CTGTGGCAGGACTTGAGGAG |
| *Lxrb* | F: GCGGACACAGAGGCAACTC  R: CTCATGACTGCACCGGCGAT |
| *Erra* | F: CACAGCCTCAGCATCTTCAA  R: ACTGCCACTGCAGGATGAG |
| *Srebp1c* | F: GGAGCCATGGATTGCACATT  R: GGCCCGGGAAGTCACTGT |
| *Cd36* | F: GCATGAATTAGTAGAACCGGGC  R: AGTTCCGATCACAGCCCATT |
| *Fabp1* | F: AGGGGGTGTCAGAAATCGTG  R: GTCATGGTCTCCAGTTCGCA |
| *Fabp3* | F: GACGGAGGCAAACTCATCCA  R: CACCACACTGCCATGAGTGA |
| *Fabp4* | F: CATAACCCTAGATGGCGGGG  R: CCAGCTTGTCACCATCTCGT |
| *Fabp5* | F: AGAGCACAGTGAAGACGACTG  R: CTTGGAAGGTGCAGACCGT |
| *Scara1* | F: GTGCTGTCTTCTTTACCAGCAA  R: GCTGTCATTGAACGTGCGTC |
| *Scarb1* | F: TGATGGAGAGCAAGCCTGTG  R: AGGATCTCACCAACTGTGCG |
| *Scarb2* | F: CCTGCTCAGGGAGCTTATCG  R: GTTCGTGCACGGTGTGAATC |
| *Fatp1* | F: GGCAAGCTCCAGCACAGGAT  R: GTCCACGGAAGTCCCAGAAAC |
| *Fatp2* | F: CATCGTGGTTGGGGCTACTT  R: GGTACCGAAGCAGTTCACCA |
| *Fatp3* | F: CGTGCTGGCCACAGAGTT  R: ATTAGTTTCAGGGCCCGTCG |
| *Hsl* | F: GCACTACAAACGCAACGAGA  R: CAGAGACGACAGCACCTCAA |
| *Lipa* | F: AATTTTGCTTCAGGCCCGCTAC  R: GCGCAAAGCTCCTTCATGATGA |
| *Lpl* | F: ACTCGCTCTCAGATGCCCTA  R: TTGTGTTGCTTGCCATTCTC |
| *Gpat* | F: GGTGAGGAGCAGCGAGATT  R: GGACAAAGATGGCAGCAGAG |
| *Mogat1* | F: TCTGGTTCTGTTTCCCGTTG  R: ACATTGCCACCTCCATCCTT |
| *Dgat1* | F: TAGAAGAGGACGAGGTGCGA  R: GTCTTTGTCCCGGGTATGGG |
| *Dgat2* | F: ACTGGAACACGCCCAAGAAA  R: GTAGTCTCGGAAGTAGCGCC |
| *Atgl* | F: CTGATGACCACCCTTTCCAA  R: TGCTACCCGTCTGCTCTTTC |
| *Pnpla3* | F: CAGGTTTGTGCCCGAATGAC  R: TGTTAAGGCTGTCACCACCC |
| *Slc25a1* | F: GGCTGTCAGGTTGGGGATGT  R: TGGGCATCCCGCATGT |
| *Acly* | F: GCCAGCGGGAGCACATC  R: CTTTGCAGGTGCCACTTCATC |
| *Acaca* | F: GGCAGCTCTGGAGGTGTATG  R: TCCTTAAGCTGGCGGTGTT |
| *Acacb* | F: GAACTCCCTGCCAAGCTCAT  R: GGTTTGGAGTTCTGGGGAGG |
| *Fasn* | F: TGAGCACACTGCTGGTGAAC  R: CAGGTTCGGAATGCTATCCA |
| *Cpt1a* | F: ACGGAGTCCTGCAACTTTGT  R: GTACAGGTGCTGGTGCTTTTC |
| *Cpt1b* | F: CCAATCATCTGGGTGCTGG  R: AAGAGACCCCGTAGCCATCA |
| *Cpt1c* | F: GGCTGGCATTGGTCAGAATC  R: CGTGCAACCTCAGGAAGTC |
| *Acox1* | F: CTCTCTATGGGATCAGCCAGAA  R: CCACTCAAACAAGTTTTCATACACA |
| *Acox3* | F: TTGAGAAGATCTATAGCCTGGAGATTT  R: AGTTCGGTGAGAGCAAAACAGC |
| *Acads* | F: AGGTTAAGAAGATGGGTGAGCTCG  R: ATGGAGTAGGCCAGGTAATCCAAG |
| *Acadsb* | F: CCCAACCTGCTTGTCTCCTTG  R: ATCCCTGGATCACCGATTTCT |
| *β-Actin* | F: CTGGGACGACATGGAGAAGA  R: ACCAGAGGCATACAGGGACA |
